# The effects of synbiotic supplementation on enteral feeding tolerance, protein homeostasis, and muscle wasting of critically ill adult patients: a randomized controlled trial

**DOI:** 10.1186/s13063-022-06668-0

**Published:** 2022-10-04

**Authors:** Najmeh Seifi, Reza Rezvani, Alireza Sedaghat, Mohsen Nematy, Majid Khadem-Rezaiyan, Mohammad Safarian

**Affiliations:** 1 Department of Nutrition, School of Medicine, Mashhad University of Medical Sciences, Mashhad, Iran; 2 Department of Nutrition, Varastegan Institute for Medical Sciences, Mashhad, Iran; 3 Department of Anesthesiology, Mashhad University of Medical Sciences, Mashhad, Iran; 4 Metabolic Syndrome Research Center, Mashhad University of Medical Sciences, Mashhad, Iran; 5 Department of Community Medicine, School of Medicine, Mashhad University of Medical Sciences, Mashhad, Iran

**Keywords:** Synbiotic, Critical illness, Enteral tolerance, Energy homeostasis, Nitrogen balance, Muscle wasting

## Abstract

**Background:**

Enteral feeding intolerance, energy-protein malnutrition, and muscle wasting are common conditions in the critical care setting. The primary aim of this study was to investigate the effect of synbiotic supplementation on enteral feed volume, energy and protein homeostasis, and muscle mass maintenance in critically ill adult patients.

**Methods:**

A consecutive of 42 patients admitted to the Edalatian Medical ICU, requiring enteral nutrition (EN), were prospectively randomized to receive the synbiotic capsule (containing a combination of *Lactobacillus*, *Bifidobacterium*, *Streptococcus*, and fructooligosaccharides) or placebo (21 patients in each group) for a maximum of 14 days. Enteral intolerance and energy homeostasis were evaluated on a daily basis. Nitrogen balance and 24-h urine creatinine excretion were recorded on days 1 and 14. Mid-arm circumference was recorded every 3 days.

**Results:**

Mean EN volume, energy, and protein intake per day were 962.5 ± 533.82 ml, 770 ± 427.05 kcal, and 38.5 ± 21.35 g (fourth day) vs. 590 ± 321.1 ml, 472 ± 256.81 kcal, and 23.6 ± 12.84 g (first day) in the synbiotic group (*p* < 0.05). Changes in the placebo group were not statistically significant. On day 1, nitrogen balance (NB) was − 19.84 ± 8.03 in the synbiotic vs. − 10.99 ± 9.12 in the placebo group (*p* = 0.003). On day 14, NB was − 14.18 ± 13.05 in the synbiotic and − 9.59 ± 7.71 in the placebo group (*p* = 0.41). Mid-arm circumference (MAC), 24-h urine creatinine, and creatinine-height index were almost steady in the synbiotic group, while they decreased in the placebo group.

**Conclusion:**

Overall, it can be concluded that enteral nutrition supplemented with synbiotics has no statistically significant effect on energy and protein homeostasis and muscle mass maintenance of critically ill patients on day 14, but it can increase enteral feed volume and energy and protein intake during the first 4 days of ICU admission.

**Trial registration:**

The trial protocol has been approved in Iranian Registry of Clinical Trials on March 17, 2019. The registration reference is IRCT20190227042857N1.

## Introduction

The majority of patients in the intensive care unit (ICU) are admitted due to severe acute illness like sepsis, severe trauma, or major surgery. Therefore, they cannot meet their nutritional needs accordingly, and this leads to energy-protein malnutrition and breakdown of muscle mass [1, 2].

Enteral feeding intolerance (EFI), a major contributor to malnutrition, is a common problem among ICU patients as it affects approximately one-third of ICU patients [3, 4]. Several pathophysiological mechanisms contribute to EFI in critically ill patients, including alteration of hormonal and/or nervous system pathways, multiple drug delivery such as sedations and broad-spectrum antibiotics, inflammation, and biochemical disturbances [5].

Gut microbiota, the neglected endocrine organ, performs many crucial protective and metabolic functions which can affect enteral feeding tolerance [6]. Gut microbiota can directly act on gastrointestinal (GI) smooth muscle contraction and mucosal absorption and secretion [7, 8]. It can also modulate neuronal and hormonal pathways which ultimately regulate glucose homeostasis, appetite, and feeding performance [9, 10]. It is also proposed that gut microbiota can influence protein balance and muscle mass maintenance independent of enteral tolerance and feed volume. Different theoretical mechanisms have been described for the gut-muscle axis which still needs to be identified [11, 12].

Considering the established extreme dysbiosis in critically ill patients [13] and the importance of malnutrition and muscle wasting in this setting, we aimed to evaluate the effect of gut microbiota modulation through synbiotics on enteral feeding tolerance, protein homeostasis, and muscle wasting of critically ill adult patients. We hypothesized that synbiotic supplementation can improve feeding tolerance, protein homeostasis, and muscle maintenance. To our knowledge, this is the first human clinical trial to evaluate the synbiotic supplementation effect on muscle wasting of critically ill patients.

## Materials and methods

This triple-blind randomized controlled clinical trial was conducted at the Edalatian intensive care unit of Imam Reza Hospital in Mashhad, Iran. The Research Ethics Committee of the School of Medicine at Mashhad University of Medical Sciences approved the study protocol (code: IR.MUMS.MEDICAL.REC.1397.715), and the research has been registered at the Iranian Registry of Clinical Trials (No. IRCT20190227042857N1). The full protocol of this study is previously published [14]. Here, a brief description is provided.

### Inclusion and exclusion criteria

ICU admitted, adult patients aged 18–65 years were included in the study if they or their guardian provided informed written consent and fulfilled the following criteria: having stable hemodynamics within 24–48 h after admission, requiring enteral nutrition (EN) via a nasogastric tube (NGT), and not receiving any microbial cell preparations (pre-, pro-, and synbiotic) during past 3 months.

Patients were excluded if they were pregnant and lactating; having any contraindications for EN or the placement of NGT; receiving immunosuppressive treatments, radiotherapy, or chemotherapy; and any of the following conditions: current renal failure, cancer, or autoimmune diseases, known allergies to microbial cell preparations, transplants receiving, hematological diseases, acquired immunodeficiency syndrome, and congenital heart valve disease or artificial heart valve.

Patients were also excluded from the final analysis if they have received the study intervention for less than 4 days.

### Product, dosage, and administration

Initially, an online stratified sequential randomization was performed based on disease severity (APACHE II scores: 0–35 and 35–70). The researchers, health care staff, subjects, and data analyzer were blinded to the procedures until the completion of the analysis. The secretary of the ICU ward was aware of randomization details. So, in case of any complications potentially attributed to the intervention, the medical staff could refer to her.

Patients in both groups received hospital gavage through NGT every 3 h (from 6 A.M. to the midnight). The same size NGTs were used for all patients and the placement was confirmed. LactoCare synbiotic capsules (500 mg; Zist Takhmir, Iran) were administered to the synbiotic group every 12 h. Each capsule contains *Lactobacillus casei* (1.5×109 CFU), *Lactobacillus acidophilus* (1.5×1010 CFU), *Lactobacillus rhamnosus* (3.5×109 CFU), *Lactobacillus bulgaricus* (2.5×108 CFU), *Bifidobacterium breve* (1×1010 CFU), *Bifidobacterium longum* (5×108 CFU), *Streptococcus thermophilus* (1.5×108 CFU), and fructooligosaccharides. The capsule was diluted in 5 ml of water and administered via the NGT, separately via gavage after feeding. The patients in the control group received a placebo capsule (Zist Takhmir, Iran), which only contained sterile maize starch which was similar to the synbiotic capsules, even in the liquid form. The intervention was continued for a maximum of 14 days in both groups.

### Outcome measurement

As previously described in the study protocol [14], the primary outcomes of this study were energy homeostasis, nitrogen balance, muscle wasting, lipolysis, glucose homeostasis, inflammatory status, and serum endotoxin level.

The secondary outcomes were enteral feed intolerance, clinical prognosis, nutritional risk, infectious complications, pressure ulcer incidence and grade, ventilation days, hospital and ICU length of stay, and mortality.

Due to multicity of outcomes and variables, here, we focused on energy homeostasis, nitrogen balance, muscle wasting, nutritional risk, and enteral feed intolerance. Other outcomes are described elsewhere [15, 16].

### Nutritional risk

To quantify the nutritional risk of critically ill patients, we used the modified NUTRIC score. A score above 5 was considered as high risk.

### Enteral feed intolerance

At each time of bullous delivery of hospital gavage, patients were evaluated for signs or symptoms of enteral intolerance. If there was vomiting, diarrhea, abdominal distention, or gastric residual volume (GRV) of more than 250 ml, feeding intolerance was recorded. Diarrhea was defined as at least 3 unformed stool episodes each day; and any regurgitation irrespective of the amount was considered as vomiting. The frequency of feeding intolerance records, the mean volume of received gavage, and prokinetic drug administration were considered as EFI. As the way enteral feed is delivered plays role in enteral feed tolerance, we educated the contributing nurses about bed angle and rate of administration before initiation of the study.

### Energy homeostasis

Energy requirement was estimated based on the simple weight-based equation of 25 Kcal/kg/day. For day1, it was estimated that the patient needs to receive 30% of the estimated calorie. Each day, we increased the estimated calorie requirement by 10%. As our previous data showed that 1 ml of our hospital gavage provides 0.8 kcal of energy, we could calculate the total received calorie (Table 1).

**Table 1:** Estimated and received energy calculations

Day	Estimated energy requirement (kcal)	Received energy (kcal)
1	(25kcal × weight) × 30%	Received enteral volume × 0.8
2	(25kcal × weight) × 40%	Received enteral volume × 0.8
3	(25kcal × weight) × 50%	Received enteral volume × 0.8
4	(25kcal × weight) × 60%	Received enteral volume × 0.8
5	(25kcal × weight) × 70%	Received enteral volume × 0.8
6	(25kcal × weight) × 80%	Received enteral volume × 0.8
7	(25kcal × weight) × 90%	Received enteral volume × 0.8
8–14	(25kcal × weight)	Received enteral volume × 0.8

### Nitrogen balance (NB)

NB was calculated by subtracting the total nitrogen (N) output from the total N intake. Total N intake was determined by dividing total daily protein intake (gram) by 6.25. The protein content of enteral gavage was 4 gr per 100 ml. If patients received supplemental parenteral nutrition, parenteral protein intake was also calculated based on the amount of amino acids. A sample of 24-h urine collection was transferred to the laboratory to measure urinary urea nitrogen (UUN) by standard enzymatic method on days 1 and 14. UUN plus 4 (skin and feces losses of N) was subtracted from total N intake [17, 18].

$$NB=\left[\ \mathrm{total}\ \mathrm{protein}\ \mathrm{intake}\ \left(\mathrm{gr}\right)\div 6.25\right]-\left(24h\ \mathrm{UUN}+4\right)$$

### Muscle wasting

To assess total body skeletal muscle, we used 24-h urine creatinine (Cr) excretion method [19, 20]. Twenty-four-hour urine collection was performed on days 1 and 14. Urine volume was recorded and an aliquot was instantly transferred to the laboratory to determine the Cr excretion by an autoanalyzer that used the Jaffe reaction. Based on previous evidence, to estimate muscle mass the following equilibrium was used [21].

$$\mathrm{Muscle}\ \mathrm{mass}\ (Kg)=\left[14.4\times 24h\ \mathrm{urinary}\ \mathrm{Cr}\ \mathrm{excretion}\ (gr)\right]+3.6$$

As 24-h urine Cr is affected by anthropometric characteristics such as height, the 24-h urine Cr/height (CHI) was evaluated as an index of muscle and protein status [22].

Mid-arm circumference (MAC), the midway between acromion, and olecranon processes of the left arm was measured 5 times during the study. All patients were lying with their arm beside. Where possible, measurements were taken on the right side of the body by the same researcher. The average of two measurements at each point were recorded.

### Statistical analysis

To achieve a statistical power of 80% with a two-sided significance level of 0.05, we needed to enroll at least 36 patients. As we estimated a drop rate of 15%, we registered 21 patients in each group of study.

Data analysis was performed in SPSS version 16 with an intent-to-treat principle. Independent and paired *t*-test, Mann-Whitney *U* test or Wilcoxon test, and repeated measures for the quantitative data and chi-square or Fisher’s exact test for the qualitative data were applied. The reported *p*-values were two-tailed, and the *p*-value of less than 0.05 was considered statistically significant.

## Results

**Fig. 1:**
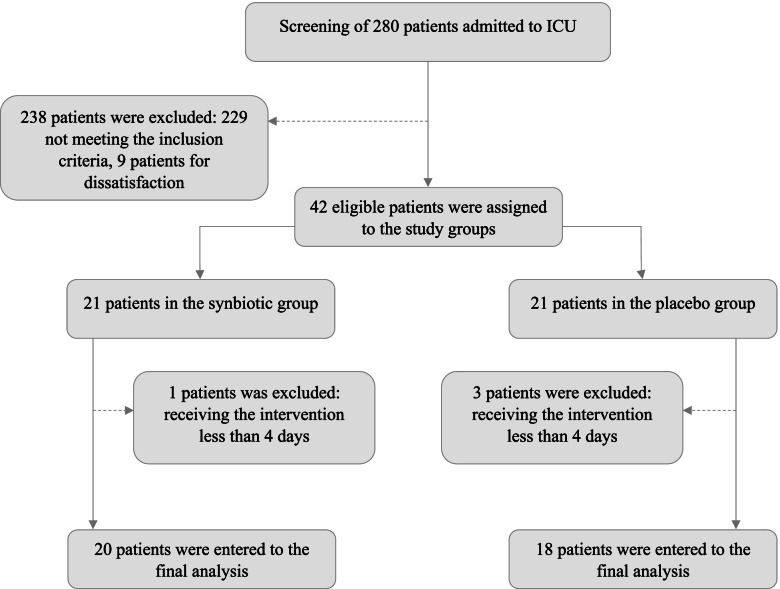
CONSORT flow diagram

Eligibility was determined in 280 ICU patients from April to October 2019, and 38 eligible patients were enrolled in the study (Fig. 1). Table 2 shows the demographic characteristics of the participants. No significant differences were observed in the baseline characteristics of the patients in the intervention and control groups.

**Table 2:** Demographic and baseline characteristics of patients

	Synbiotics (***n*** = 20)	Placebo (***n*** = 18)
**Mean age (year)**	38.5 ± 17.94	47.61 ± 22.51
**Gender** *N* (%)		
Male	9 (45)	14 (78)
Female	11 (55)	4 (22)
**Smoking habits** *N* (%)		
Previously	10 (50)	12 (66.7)
Currently	9 (45)	10 (55.5)
**Past medical history** *N* (%)		
Diabetes	0	3 (16.6)
CVD	0	3 (16.6)
Neurological disorders	3 (15)	3 (16.6)
Psychological disorders	5 (25)	4 (22.2)
Cause of ICU admission *N* (%)		
Intoxication	12 (60)	10 (55.5)
Pulmonary	3 (16.7)	1 (5)
Cardiac	0	3 (15)
Neurologic	1 (5.6)	1 (5)
Sepsis	3 (16.7)	2 (10)
Others	1 (5.6)	1 (5)
Disease severity (APACHEII) Mean ± SD	17.40 ± 4.72	18 ± 4
Duration of presence in the study	9.45 ± 4.43	11.22 ± 3.68

*CVD* cerebrovascular disorders, *ICU* intensive care unit, *APACHEII* Acute Physiology and Chronic Health Evaluation

As it is shown in Table 3, the NUTRIC score on days 1, 7, and 14 was less than 5 (low nutritional risk), and there was no statistically significant difference between the two groups. EN start day, EN duration, and EN days per ICU stay days were similar in both groups. In the synbiotic group, 3 patients (15%) needed supplemental PN during the study (*p* **=** 0.99); in the placebo group, it was 2 patients (11.1). Mean EN volume per day in the synbiotic and placebo groups was 1313.6 ± 417.56 ml and 1315.2 **±** 565.66 ml, respectively (*p* **=** 0.79). The mean energy deficit was about 300 kcal in both groups (*p* **=** 0.92). EFI was recorded 3.5 times in the synbiotic and 6.77 times in the placebo group. The difference was not statistically significant. Prokinetics administration frequency and duration were similar in both groups.

**Table 3:** Nutritional status, enteral feed volume, and energy homeostasis in the synbiotic and placebo groups

	Synbiotics (***n*** = 20)	Placebo (***n*** = 18)	***p***-value
**NUTRIC score**			
Day 1	2.7 ± 1.4	3.61 ± 2.25	0.28^a^
Day 7	2.42 ± 2.02	3.93 ± 2.40	0.07^b^
Day 14	3.08 ± 2.06	3.63 ± 2.29	0.54^b^
**EN start day**	2.85 ± 1.89	2.33 ± 1.41	0.36^a^
**EN duration (days)**	8.85 ± 4.41	10.44 ± 1.41	0.36^a^
**EN days per ICU stay days (%)**	93.52 ± 12.67	94.29 ± 14.07	0.59^a^
**Supplemental PN** ***N*** **(%)**	3 (15)	2 (11.1)	0.99^a^
**Mean EN volume per day (ml)**			
1st week	940.27 ± 424.72	976.50 ± 714.88	0.67^b^
2nd week	1507.7 ± 495.85	1561.1 ± 480.20	0.77^b^
Total	1313.6 ± 417.56	1315.2 ± 562.66	0.79^b^
**Energy homeostasis (Kcal)**			
1st week	− 205.3 ± 308.9	− 214.3 ± 642.39	0.95^b^
2nd week	− 446.7 ± 274.8	− 428.6 ± 512.26	0.91^b^
Total	− 271.4 ± 262.36	− 289.8 ± 565.21	0.92^b^
**EFI records frequency**	3.5 ± 4.01	6.77 ± 3.77	0.85^a^
**Prokinetics administration,** ***N*** **(%)**	3 (15)	3 (16.7)	0.99^c^
**Prokinetics administration duration (days)**	6 ± 3.6	3.75 ± 3.77	0.27^a^

*EN* enteral nutrition, *EFI* enteral feeding intolerance, *ICU* intensive care unit, *NUTRIC* nutrition risk in critically ill, *N* number, *PN* parenteral nutrition

^a^Mann-Whitney *U* test

^b^Independent *t* test

^c^Fisher’s exact test

As it is demonstrated in Table 4, protein intake during the first week, the second week, and all study duration was not significantly different between the two groups. Nitrogen balance was negative on days 1 and 14 in both study groups. On day 1, it was − 19.84 in the synbiotic and − 10.99 in the placebo group (*p* = 0.003). On day 14, it raised to − 14.18 in the synbiotic and − 9.59 in the placebo group (*p* = 0.41). To compare NB changes within each group, our results showed that the mean difference was 11.75 ± 6.96 (*p* = 0.15) in the synbiotic group and 1.39 ± 2.09 (*p* = 0.5) in the placebo group.

**Table 4:** Protein intake and nitrogen balance in the synbiotic and placebo groups

	Synbiotics (***n*** = 20)	Placebo (***n*** = 18)	***p***-value
**Protein intake (g)**			
1st week	37.61 ± 16.98	39.06 ± 28.59	0.85^a^
2nd week	60.3 ± 19.83	62.44 ± 19.20	0.79^a^
Total	52.54 ± 16.7	52.60 ± 22.5	0.99^a^
**Protein intake per body weight (g/kg)**			
1st week	0.78 ± 0.2	0.81 ± 0.41	0.82^a^
2nd week	0.58 ± 0.24	0.62 ± 0.48	0.78^a^
Total	0.9 ± 0.23	0.96 ± 0.37	0.64^a^
**Nitrogen balance (g)**			
Day 1	− 19.84 ± 8.03	− 10.99 ± 9.12	0.003^b^
Day 14	− 14.18 ± 13.05	− 9.59 ± 7.71	0.41^b^

^a^Mann-Whitney *U* test

^b^Independent *t* test

**Fig. 2:**
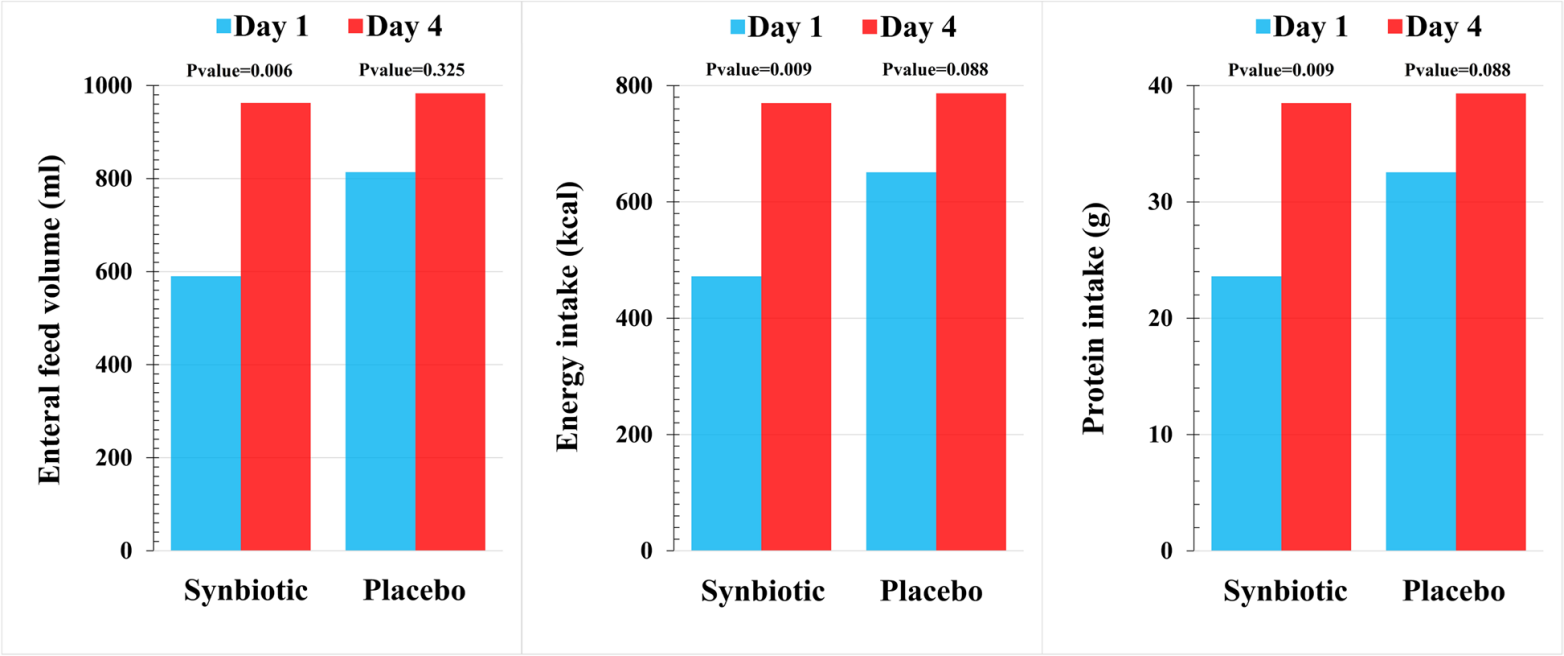
Enteral feed volume and energy and protein intake on days 1 and 4 in the synbiotic and placebo groups

As Fig. 2 shows, enteral feed volume, calorie, and protein intake significantly increased from day 1 to day 4 in the synbiotic group, while the increase in the placebo group was not statistically significant.

**Fig. 3:**
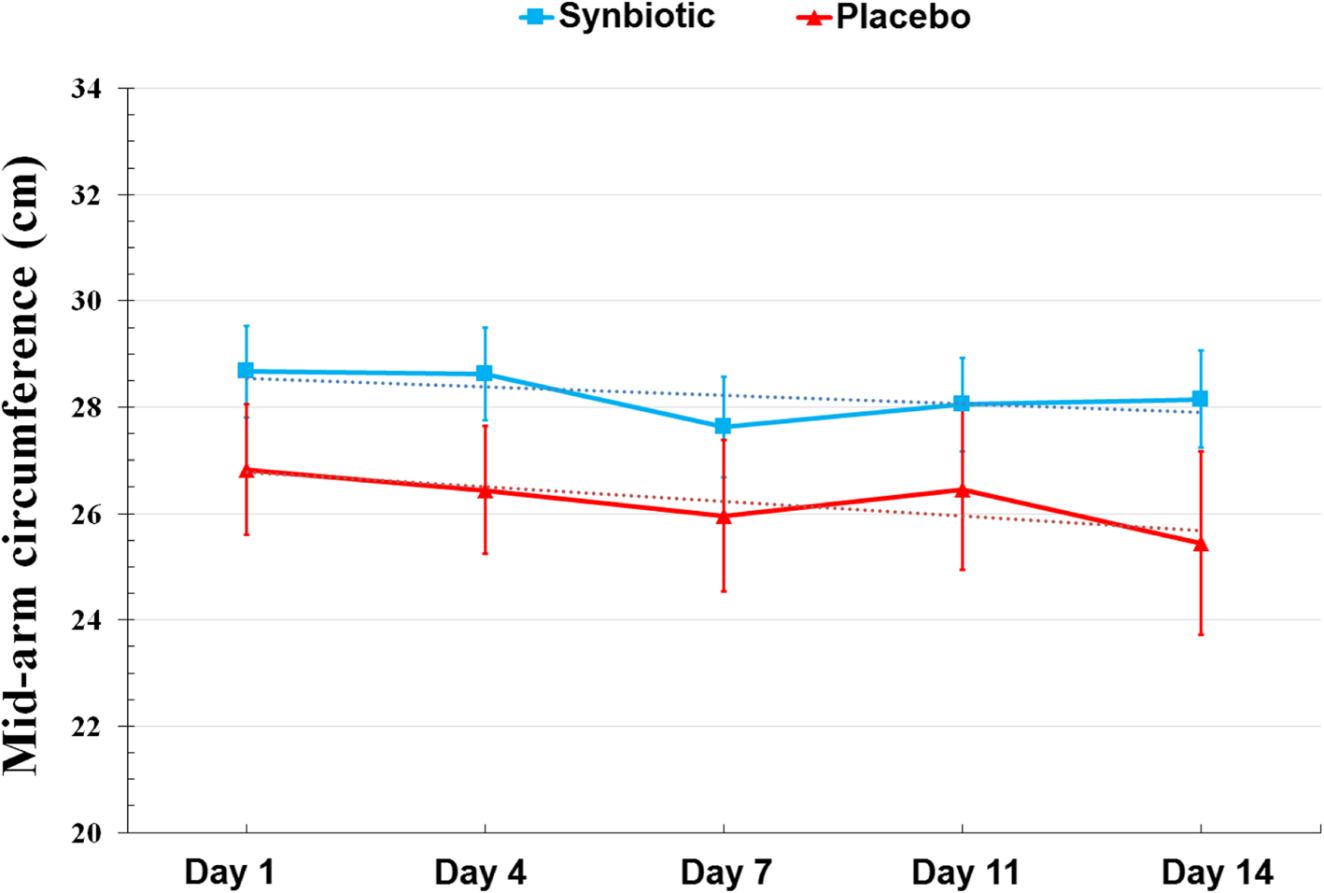
Mid-arm circumference changes during the study period

Mid-arm circumference measurements during the study period are presented in Fig. 3. In the placebo group, there was a significant linear reduction of MAC during the study (*p* < 0.001). But, in the synbiotic group, changes were not statistically significant (*p* = 0.19).

As it is shown in Table 5, 24-h urine Cr and CHI decreased in the placebo group and was almost steady in the synbiotic group. Muscle mass also showed a 3-kg decrease in the placebo group, while it did not change in the synbiotic group. Although the differences are not statistically significant, they are clinically remarkable.

**Table 5:** Muscle mass status of synbiotic and placebo groups on days 1 and 14

Variable	Synbiotic group			Placebo group			***p***-value day 1^**d**^	***p***-value day 14^**d**^
	Day1	Day 14	***p***-value^**c**^	Day1	Day14	***p***-value^**c**^		
MAC (cm)	28.75 ± 2.60	28.15 ± 2.93	0.17^1^	26.60 ± 5.52	25.45 ± 5.44	0.001^a^	0.22^b^	0.18^b^
24h urine Cr (mg)	863.56 ± 348.66	867.11 ± 407.85	0.98^1^	795.72 ± 585.76	648.44 ± 242.29	0.39^a^	0.96^b^	0.18^b^
CHI (%)	67.35 ± 27.66	64.33 ± 76.01	0.86^1^	60.39 ± 68.56	52.22 ± 59.77	0.53^a^	0.91^b^	0.2^b^
Muscle mass (Kg)	16.03 ± 5.02	16.08 ± 5.87	0.98^1^	15.05 ± 8.43	12.93 ± 3.48	0.39^a^	0.96^b^	0.18^b^

Data are reported as standard deviation ± mean

*CHI* creatinine height index, *Cr* creatinine, *MAC* mid-arm circumference

^a^Paired sample *t* test

^b^Independent *t* test

^c^Comparison within the groups

^d^Comparison between the groups

## Discussion

In this study, we set out to investigate the effect of synbiotic supplementation on enteral feeding intolerance, protein status, and muscle maintenance of critically ill adult patients. The results of this study suggest that synbiotic are associated with a higher intake of hospital gavage, energy, and protein during the first 4 days, nitrogen balance improvement, and muscle mass maintenance in the synbiotic group.

Although enteral feed volume, energy, and protein intake increased significantly during the first 4 days in the synbiotic group, the overall changes were not significant, and there was no difference in the total EN volume and energy or protein intake between the two groups. Our recent systematic review also showed that synbiotic, probiotic, or prebiotic does not affect energy intake and feed volume in tube-fed critically ill patients [23]. Although, Rushdi et al. showed that prebiotic supplementation was associated with significantly increased feed volume on day 4 in the intervention group [24], which was inconsistent with our results.

Delayed gastric emptying and feeding intolerance are common in the critical care setting [25]. EFI usually occurs during the first few days after the start of EN (specifically on the 3rd day). The underlying mechanisms are not well-known, but it seems that elevated level of CCK and PYY and their increased response to the presence of the small amount of nutrients in the gut, inflammation, hyperglycemia, variety of medications, as well as gut microbiota dysbiosis, are among the involved mechanisms [26, 27]. Dysbiosis occurs within a few hours of ICU admission [28], and its effect on feeding intolerance becomes evident during the first few days. Therefore, synbiotic supplementation efficacy through its effect on gut muscle contractions, secretion and absorption, glucose homeostasis and neuro-hormonal regulations, appetite, and feeding behavior [7, 9–11] is more related to the first few days of enteral feeding.

Nitrogen balance was significantly more negative in the synbiotic group on day 1. But, on day 14, the difference between the two groups was not statistically significant. Although the improvement of nitrogen balance was not statistically significant in either group, it was clinically remarkable in the synbiotic group. As energy and protein intake were similar in the two groups, the improvement may be attributed to the improvement of digestion and absorption of proteins in the GI tract. Some previous studies showed that probiotics can affect digestion and absorption of proteins through different mechanisms including activation of digestive protease and peptidase, improvement of absorption of small peptides and aminoamides, and reduction of harmful protein fermentation. In contrast to our results, Falco de Arruda et al. showed that enteral nutrition supplemented with probiotics and glutamine was not associated with nitrogen balance improvement in head trauma patients [29].

The results of this study showed that MAC in the synbiotic group remained almost constant during the study, while it significantly decreased in the placebo group. Twenty-four-hour urine Cr and CHI, as indicators of muscle mass, also remained constant in the synbiotic group, while they decreased in the placebo group. Although this decrease was not statistically significant, the reduction of 2 kg of muscle mass in the placebo group seems to be clinically remarkable. Numerous animal studies support the effect of gut microbiota homeostasis on muscle mass maintenance [30–33], but no human study have examined this effect. According to the limited available studies, it seems that gut microbiota affects muscle pathophysiology through different mechanisms including maintenance of gut barrier function, reduction of endotoxin translocation and inflammation, improvement of insulin sensitivity, mitochondrial biogenesis, muscle anabolism, and reduction of myocyte apoptosis by important metabolites such as short- chain fatty acids (SCFAs), regulation of amino acids bioavailability for muscle protein synthesis, anabolism stimulation and oxidative stress suppression by synthesis of Vit B group, glycine, and betaine [34, 35].

To our knowledge, this is the first human clinical trial investigating the effect of synbiotic supplementation on muscle wasting in critically ill patients, although there are some limitations. We feed our patients with hospital gavage which contains meat protein. If we could use standard commercial gavage, we could also measure 24-h urine 3-methyl- Histidine (3MH), which is exclusively found in the skeletal muscle, and its urinary excretion shows muscle protein degradation [36]. Also, due to executive restrictions, we were not able to increase the enteral feed volume to the patients’ tolerable level, and we obeyed the enteral nutrition policy of the ICU ward. Although their policy is to gradually increase EN in case of tolerance and decrease in case of intolerance, our results cannot lead to an exact conclusion about the enteral feed tolerance of our patients. Another limitation in this study was the presence of multiple primary outcomes without taking steps to adjust for the performance of multiple statistical comparisons. Finally, we suggest further clinical trials that determine gut microbiota changes by molecular techniques.

## Conclusion

The results of this study demonstrated that enteral nutrition supplemented with synbiotics containing a combination of *Lactobacillus*, *Bifidobacterium*, *Streptococcus*, and fructooligosaccharides had no statistically significant effect on energy and protein homeostasis and muscle mass maintenance of critically ill patients on day 14, but it can increase enteral feed volume and energy and protein intake during the first 4 days of ICU admission.

## Data Availability

The datasets generated and/or analyzed during the current study are not publicly available due to ethical considerations but may be available from the corresponding author on reasonable request.

## Abbreviations

A.M.: Ante meridiem
APACHE II: Acute Physiology and Chronic Health Evaluation
CCK: Cholecystokinin
CFU: Colony-forming unit
CHI: Creatinine Height Index
Cr: Creatinine
CVD: Cerebrovascular disorders
EFI: Enteral feeding intolerance
EN: Enteral nutrition
GI: Gastrointestinal
GRV: Gastric residual volume
ICU: Intensive care unit
MAC: Mid-arm circumference
N: Nitrogen
NB: Nitrogen balance
NGT: Nasogastric tube
NUTRIC: Nutrition risk in critically ill
PN: Parenteral nutrition
PYY: Peptide YY
SCFA: Short-chain fatty acids
UUN: Urinary urea nitrogen
3MH: 3-methyl-histidine
